# Inflammatory, Serological and Vascular Determinants of Cardiovascular Disease in Systemic Lupus Erythematosus Patients

**DOI:** 10.3390/ijms20092154

**Published:** 2019-04-30

**Authors:** Valentina Mercurio, Antonio Lobasso, Letizia Barbieri, Paolo Parrella, Deasy Ciervo, Bianca Liccardo, Domenico Bonaduce, Carlo G. Tocchetti, Amato De Paulis, Francesca W. Rossi

**Affiliations:** 1 Department of Translational Medical Sciences, Federico II University, 80131 Naples, Italy; valemercurio@yahoo.com (V.M.); antonio_lobasso@libero.it (A.L.); letiziabarbieri@yahoo.it (L.B.); paoloparrella01@libero.it (P.P.); deasy@live.it (D.C.); biandom@libero.it (B.L.); bonaduce@unina.it (D.B.); frawrossi@yahoo.it (F.W.R.); 2 Center for Basic and Clinical Immunology Research (CISI), WAO Center of Excellence, 80131 Naples, Italy

**Keywords:** cardiovascular disease, systemic lupus erythematosus, inflammation, arterial stiffness, augmentation index

## Abstract

Background and aim: Systemic lupus erythematosus (SLE) is associated with increased risk of cardiovascular disease (CVD). Among many mechanisms, accelerated atherosclerosis, endothelial dysfunction, and hypercoagulability play a main role. Here, we investigate whether inflammatory, serological and clinical markers of SLE determine and correlate with arterial stiffness in SLE patients. Materials and methods: Routine blood samples, inflammatory mediators, specific antibodies, and 24 h proteinuria were measured in 43 SLE patients and 43 age and sex-matched controls using routine laboratory assays. We also assessed arterial stiffness by measuring radial artery applanation tonometry-derived augmentation index (AI), normalized AI (AIx@75), aortic pulse pressure, central systolic, diastolic and peripheral blood pressure. Results: SLE patients showed a significantly greater arterial stiffness vs. controls, as demonstrated by the significantly higher AIx@75 and aortic pulse pressure. Interestingly, regression analysis showed that age, systolic pulse pressure, inflammatory markers (erythrocyte sedimentation rate and C-reactive protein), daily dose of glucocorticoids, and cumulative organ damage positively correlated with arterial stiffness. Conclusions: SLE patients show increased arterial stiffness which correlates with markers of inflammation, that is involved in early alterations in arterial walls. Applanation tonometry can be used to screen SLE patients for subclinical vascular damage to implement prevention strategies for CVD.

## 1. Introduction

Systemic lupus erythematosus (SLE) is an autoimmune inflammatory disease mainly affecting women of childbearing age [1]. Cardiovascular disease (CVD) is a well-known complication of SLE, accounting for more than one third of death causes [2,3]. Compared with age and sex-matched controls, SLE patients generally show an increased incidence of CVD [4], and cardiovascular events (CVE) account for the main cause of death [5]. The exact mechanisms contributing to the increased cardiovascular risk of SLE patients are still debated. Some authors highlighted the possible implications of systemic inflammation, specific disease-related factors, and corticosteroid therapy [6,7,8,9], but relatively few data are available so far. However, it is well established that patients with SLE present an increased incidence of atherosclerosis and an accelerated progression of the atherosclerotic plaques [4,10]. SLE-related immune dysregulation and inflammatory environment seem to be crucial in the development of atherosclerosis. This hypothesis is further supported by the finding that atherogenesis-involved genes may work synergistically with autoimmune response-promoting genes [11].

Alterations in arterial distensibility and compliance have been described in chronic inflammatory diseases [12]. They may be due to underlying increased inflammation-mediated sub-clinical damage, in addition to traditional risk factors. Early in the development of atherosclerotic disease, some modifications of the mechanical properties of the arteries occur [13], and they might be considered as predictive for the risk of future CVEs. Evaluation of aortic stiffness by means of applanation tonometry of the radial artery is a simple and non-invasive technique for the quantitative assessment of subclinical vascular impairment [14]. Previous studies have demonstrated its prognostic utility, in addition to the other traditional techniques, for the risk stratification of hypertensive patients and patients with a variety of cardiovascular diseases [15,16]. Here, we aim at exploring whether in SLE patients the inflammatory status can be recapitulated by serological and inflammatory markers of disease and can translate into arterial stiffness, detectable with clinical and vascular alterations assessed by means of applanation tonometry of the radial artery.

## 2. Results

### 2.1. Patients’ Characteristics

A total of 43 SLE patients (4 males and 39 females; median age 41 (11) years) were enrolled at the Department of Translational Medical Sciences, Federico II University, Naples, between March 2018 and October 2018. All patients fulfilled the 2012 Systemic Lupus International Collaborating Clinics (SLICC) criteria [17]. Forty-three healthy subjects served as age and gender-matched controls. Eleven patients (25.5%) were in menopause (54.5% natural menopause and 45.5% iatrogenic menopause). None of the patients had metabolic syndrome.

At the time of the enrolment, 39 patients (90.7%) were on glucocorticoid therapy (median daily dose of prednisone or prednisone equivalent of 7.5 (8) mg). The median duration of the disease was 14 (18) years. SLE disease activity was assessed using the Systemic Lupus Erythematosus Disease Activity Index (SELENA-SLEDAI) score [18]. The median value of the disease activity index evaluated by SELENA/SLEDAI was 8 (10, limits 0–26). The median value of the SLICC/ACR damage index was 2 (2, limits 0–7). The most frequently observed clinical and laboratory components of SELENA-SLEDAI index were active SLE nephritis (21%), increased DNA binding (44.2%), low complement (48.8%), arthritis (44.1%), and rash (13.9%).

The median value of systolic blood pressure (SBP) was 120 (20) mmHg. Blood tests included total cholesterol, high-density lipoprotein cholesterol, low-density lipoprotein cholesterol, homocysteine, creatinine, acid uric, glucose lymphocytes, C-reactive protein (CRP), erythrocyte sedimentation rate (ESR), fibrinogen and lupus serology (anti-DNA antibodies, complement). 24-h proteinuria sample evaluated the urinary protein excretion. All the characteristics of the study population are shown in Table 1.

### 2.2. Comparison of Arterial Stiffness between Systemic Lupus Erythematosus (SLE) and Healthy Controls

**Figure 1:**
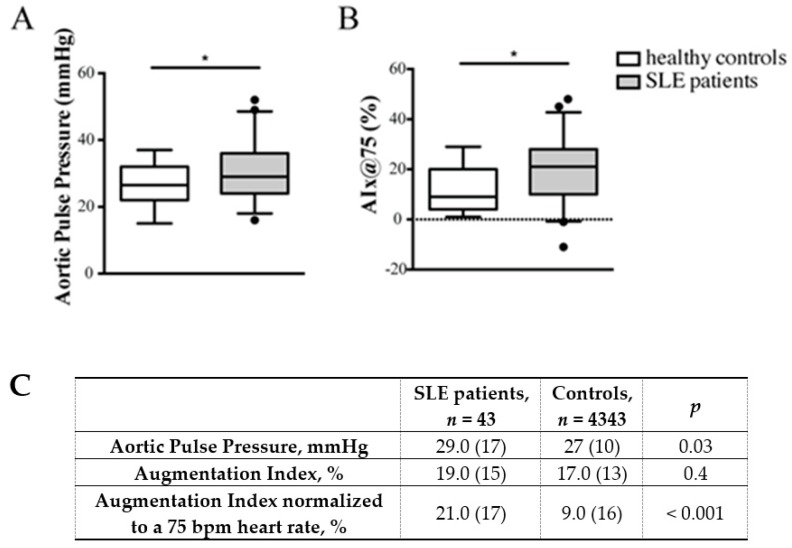
(**A**) Box plots of aortic pulse pressure (AoPP) in SLE patients and controls. SLE patients showed increased arterial stiffness compared to controls. AoPP resulted higher in SLE patients compared to controls (* *p* = 0.03). (**B**) Box plots of AIx@75 in SLE patients and controls. Higher values of AIx@75 (* *p* < 0.001) were detected in SLE patients. (**C**) Values of arterial stiffness in SLE patients and controls. List of abbreviations: SLE, Systemic Lupus Erythematosus. Outliers are shown as blank dots. The horizontal dotted line delimits zero.

SLE patients exhibited increased arterial stiffness compared to controls (Figure 1, Panels A,C). In particular, aortic pulse pressure (AoPP) was higher in SLE patients compared to controls (29.0 (17) mmHg vs. 27 (10) mmHg; *p* = 0.03). SLE patients presented significantly higher values of AIx@75 (21.0 (17) vs. 9.0 (16); *p* < 0.001) (Figure 1, Panels B,C). AIx was also higher in patients than in controls, even though the difference was not statistically different.

Reprodicibility of AoPP, AIx, and AIx@75 was excellent (intraclass coefficient correlations of 0.997, 0.981, and 0.989 for the intra-observer variability, and of 0.980, 0.939, and 0.970 for the inter-observer variability, respectively).

### 2.3. Predictors of Arterial Stiffness

**Figure 2:**
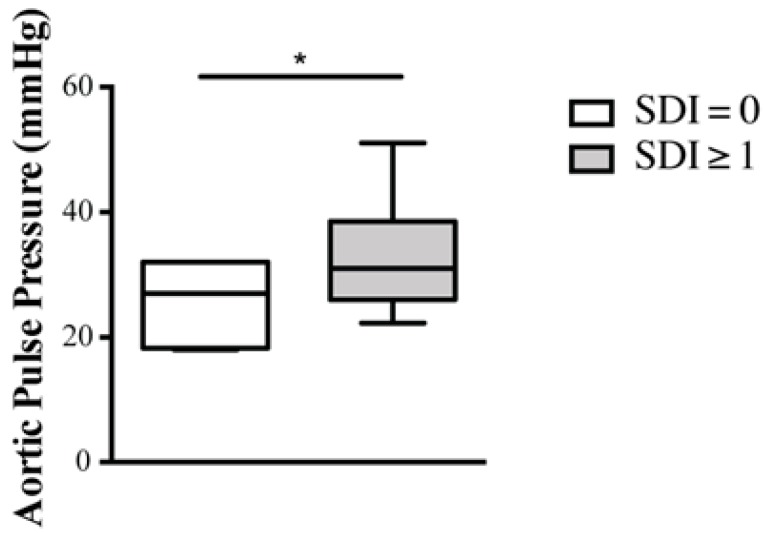
Box plots of AoPP in SLE patients with and without organ damage. Patients with organ damage, defined by a SDI score ≥ 1, showed significantly higher values of AoPP than those without no organ damage (32 (9) vs. 28 (7), * *p* = 0.03). List of abbreviations: SLE, systemic lupus erythematosus; SDI, Systemic Lupus International Collaborating Clinics/American College of Rheumatology Damage Index.

In the univariate analysis (Table 2), AoPP correlated with age (r = 0.4; *p* < 0.05), SBP (r = 0.66; *p* < 0.001), CRP levels (r = 0.37; *p* < 0.05), ESR (r = 0.34; *p* = 0.05), daily dose of prednisone (r = 0.33; *p* = 0.05) and damage index (r = 0.30; *p* = 0.05). Moreover, patients who displayed organ damage, defined by a SLICC/ACR damage index score (SDI) ≥ 1, had significantly higher AoPP than those with a SDI = 0 (32 (9) vs. 28 (7), *p* = 0.03), as shown in Figure 2.

AIx@75 showed positive correlation with age (r = 0.57; *p* < 0.001), negative correlation with lymphocyte count (r = −0.44; *p* < 0.01). AIx directly correlated with age (r = 0.59; *p* < 0.001), inversely blood creatinine levels (r = −0.37; *p* < 0.05) and lymphocyte count (r = −0.34; *p* = 0.05).

Regarding the clinical manifestations, both AIx@75 and AIx resulted significantly higher in patients with clinical manifestations, especially in patients who had mucosal ulcers (*p* = 0.03 and 0.04, respectively) and active nephritis (*p* = 0.01 and *p* = 0.006) at the time of the study.

At the multiregression analysis (Table 3), AoPP correlated with age (r^2^ = 0.53; 95% confidence interval (C.I.) 0.20 to 0.89; *p* = 0.01), SBP (r^2^ = 0.29; 95% C.I. 0.12 to 0.45; *p* = 0.001), ESR (r^2^ = 0.29; 95% C.I. 0.09 to −0.49; *p* = 0.005). Both AIx@75 and AIx correlated with age (r^2^ = 0.80 with 95% C.I. 0.33 to 1.28 and *p* < 0.001 for AIx@75; r^2^ = 0.95 with 95% C.I. 0.48 to 1.43 and *p* < 0.001 for AIx).

## 3. Discussion

CVD is an important factor for SLE mortality [7]. Importantly, SLE pathophysiology is characterized by increased incidence of atherosclerosis with accelerated progression of atherosclerotic plaques [4,10]. Hence, early prediction of CVD is very important for appropriate treatment and follow-up of SLE patients.

Previous work assessing arterial stiffness in SLE patients by means of applanation tonometry provided controversial results [19,20,21,22,23,24,25]. In our study we found significantly higher values of AIx@75 and AoPP at applanation tonometry in SLE patients compared to age and sex-matched healthy controls. Furthermore, AoPP showed a significant correlation with inflammatory markers such as ESR. In SLE, immune dysregulation and the inflammatory environment seem to be crucial in the development of atherosclerosis. CRP levels are known to be independent predictors of cardiovascular risk, and contribute directly to the genesis of atherosclerotic lesions [26], determining endothelial dysfunction [27], which is a marker of vascular damage and is a well-known feature of SLE [28]. Endothelial dysfunction is a main factor in the early development of atherosclerosis in SLE patients [29]. This is considered as a negative prognostic factor for CVD, possibly related to a specific dyslipidaemic pattern in SLE patients [30] and to the increase of inflammatory cytokines, especially type I interferons (IFNs) [31,32]. Indeed, hypercholesterolemia has been described as the main predictor of cardiovascular events (CVE) in SLE patients [33,34]. In particular, LDL-cholesterol levels have been demonstrated to be associated with impairment in the augmentation index [35]. Nevertheless, in line with previous studies [21,36] we found no correlation between cholesterol levels and AoPP. This result should be interpreted considering that none of the patients had hypercholesterolemia, defined as serum total cholesterol higher than 250 mg/dL, the threshold correlated with a significant increase of cardiovascular risk [7].

Among other traditional cardiovascular risk factors, age showed the strongest correlation with all the parameters of arterial stiffness. This is coherent with what is known in the general population, as arterial stiffness increases with age [37,38]. AoPP also showed a significant positive correlation with SBP (Table 2). These findings confirm data previously reported, as a strong association between hypertension and arterial stiffness in general population has been described [39,40]. Interestingly, in our study, SBP correlated with arterial stiffness even in normotensive patients.

On the other hand, arterial stiffness did not show a significant correlation with disease activity, assessed with the SELENA-SLEDAI 2K tool. However, the parameters of arterial stiffness were significantly higher in patients with mucosal ulcers and active nephritis. Interestingly, an association between subclinical atherosclerosis and impaired renal function has been reported in some studies on SLE patients [41,42].

One of the most debated SLE-related factors contributing to vascular stiffness is represented by glucocorticoid therapy. In our analysis, AoPP showed a significant correlation with the dose of prednisone (or equivalent). The contribution of glucocorticoids to the CV risk in SLE patients is yet to be determined, but it has to be acknowledged that patients treated with higher glucocorticoids disease are usually those with the most severe clinical phenotype. Indeed, our results show that arterial stiffness correlates with cumulative organ damage, as assessed by the SLICC/ACR damage index (SDI). AoPP was significantly higher in patients with (SDI score ≥ 1) than in those with no organ damage (Figure 2). Moreover, aortic pulse pressure had a positive correlation with the extent of organ damage, as measured by the damage index, in agreement with previous studies [43]. These findings should be further explored to determine whether arterial stiffness might act as a predictor of organ damage in SLE patients.

The impact of glucocorticoid therapy on cardiovascular risk is relevant also because of the metabolic alterations occurring with long-term treatment with steroids [33,44]. However, it has been reported that such alterations only occur with daily doses exceeding 10 mg and for therapies lasting more than 6 months [45]. Moreover, since inflammation is strongly correlated with the atherosclerotic process, low-dose corticosteroids might have a beneficial anti-inflammatory and anti-atherosclerotic action. For these reasons, corticosteroids remain beneficial treatment in SLE, but dose-tapering should be started as soon as possible, depending on the clinical status of the patient. Glucocorticoid tapering should be encouraged as soon as disease activity allows it, preferring combination therapy [46]. Yet, our study highlights that some therapy related-risk may exist.

## 4. Materials and Methods

### 4.1. Study Design and Ethics Statement

In our study we included 43 subjects older than 18 years who fulfilled the 2012 Systemic Lupus International Collaborating Clinics (SLICC) criteria [17], treated, at enrolment, with prednisone at a stable dose for at least 3 months. Exclusion criteria were: pregnancy, history of cardiovascular events or known coronary disease, uncontrolled hypertension, the presence of arrhythmias or the presence of a pacemaker, cancer or acute infectious diseases. A full clinical history and physical examination, including weight, height, body mass index, smoking status and blood pressure, was collected. The mean disease duration, demographics, and clinical data were obtained from the medical records. Forty-three healthy subjects served as age and gender-matched controls.

The study was conducted in accordance with the principles of the 1975 Declaration of Helsinki and good clinical practice guidelines of the International Conference of Harmonization (ICH GCP). Informed consent was obtained from all patients before the procedure, and the study was approved by the local ethical committee of the University of Naples Federico II “Carlo Romano” (348/2018 approved 14 March 2018).

### 4.2. Clinical and Laboratory Data

Routine blood samples, inflammatory mediators, specific antibodies, and 24 h proteinuria were measured using routine laboratory assays. A detailed clinical history was collected, including current and cumulative use of glucocorticoids. Disease activity in the preceding 30 days was assessed with the use of the SELENA-Systemic Lupus Erythematosus Disease Activity Index (SELENA-SLEDAI-2K) [18]. Cumulative organ damage was calculated with the Systemic Lupus International Collaborating Clinic/American College of Rheumatology (SLICC/ACR) damage index (Table 1) [17].

### 4.3. Vascular Assessment

Brachial blood pressure was measured at the non-dominant arm with a sphygmomanometer. Parameters of arterial stiffness were derived by a high fidelity applanation tonometer (SphygmoCor device, AtCor Medical, Sydney, Australia). Measurements were performed in a quiet and controlled temperature room, after 10 min in supine position, with the patient fasting and refraining from alcohol and coffee assumption for at least 10 h. All measurements were made in duplicate by two trained operators (D.C., P.P.) according to the international recommendations (the values with the higher Operator Index were used in the analysis) and interpreted by a different operator (V.M.) who was blind to the clinical characteristics of the patients. Aortic pulse pressure (AoPP), Augmentation Index (AIx) and Augmentation Index normalized to heart rate of 75 bpm (AIx@75) were the parameters considered in order to evaluate arterial stiffness. In particular, AoPP was defined as the difference between the first peak of systolic blood pressure and the diastolic blood pressure; AIx was calculated as the ratio of late systolic pressure to early systolic pressure, expressed as a percentage of the sphygmic wave [47].

### 4.4. Statistical Analyses

Continuous variables were expressed as median ± interquartile range; categorical variables were expressed as frequencies or percentages. The comparison between patients’ and controls’ data was performed by non-parametric Mann–Whitney U-test for independent samples. Factors associated with arterial stiffness, in SLE patients, were analyzed by the use of Pearson’s correlation coefficient or Spearman’s correlation (univariate analysis) and multiple linear regression with OLS method (multivariate analysis). Variables correlated to stiffness with a p-value below 10% in univariate analysis were considered for the multivariate model. A *p* value ≤0.05 was considered as statistically significant.

Intra- and inter-observer variabilities for parameters of arterial stiffness were evaluated by means of intraclass correlation coefficients (10 random exams were preformed twice by an operator and compared with the results obtained by a second operator).

## 5. Conclusions

Overall, our results support the concept that inflammation is a main determinant of cardiovascular complications in SLE. As highlighted by a recent meta-analysis, SLE patients have a higher carotid intima-media thickness and an increased prevalence of carotid plaques, supporting the evidence for an increased CVD risk [9].

Our study has some limitations to be considered. The sample size is small and may lack statistical power to define additional factors correlated to arterial stiffness, especially concerning the results of the regression analysis. Moreover, a cause–effect relation cannot be inferred from a cross-sectional study. Nevertheless, our results suggest that a regular and careful serological and clinical assessment together with vascular investigations is useful to identify an early impairment of arterial stiffness in patients with SLE. Our data also support the use of non-invasive techniques such as applanation tonometry of the radial artery to assess the accelerated progression of atherosclerotic damage of the vessel wall in SLE patients. It is particularly important to stratify high-risk patients in order to plan an appropriate therapeutic approach for SLE patients and control traditional risk factors to avoid subclinical vascular impairment evolving into manifest cardiovascular events [48]. Therefore, early detection of increased aortic stiffness in these patients may be beneficial to highlight the vascular damage and to prompt an early therapeutic intervention. Eventually, this approach may attenuate the inflammatory process and prevent cardiovascular damage.

## Figures and Tables

**Table 1:** Characteristics of SLE (systemic lupus erythematosus) and controls subjects.

Characteristics	SLE Patients, *n* = 43	Controls, *n* = 43	*p*
**Age, years**	41 (11)	39.0 (12)	0.18
**Males, n (%)**	4 (9.3%)	4 (9.3%)	0.10
**Systolic blood pressure, mmHg**	120 (20)	118 (15)	0.334
**Diastolic blood pressure, mmHg**	80 (15)	8 (10)	0.225
**Body mass index, kg/m^2^**	23.2 (6.0)	23.0 (6)	0.720
**Hypertension, n (%)**	7 (16.3%)	6 (13.9%)	0.422
**Type 2 diabetes mellitus, n (%)**	1 (2.5%)	0	0.225
**Smoking habit, n (%)**	21 (48.8%)	18 (41.9%)	0.633
**Disease duration, years**	14.0 (18)		
**Duration of steroid therapy, years**	10 (13)		
**Mean daily dose of prednisone, mg**	7.5 (8)		
**Diagnosis of nephritis, n (%)**	9 (22.5%)		
**Mean SLEDAI score**	8.0 (10)		
**Mean SDI score**	2 (2)		
**ANA**	37 (86%)		
**anti-dsDNA**	19 (44.2%)		
**ENA**	25 (58.1%)		
**Low complement levels (C3 or C4)**	21 (48.8%)		
**Total cholesterol, mg/dL**	174 (75)		
**LDL cholesterol, mg/dL**	112 (53)		
**HDL cholesterol, mg/dL**	52 (17)		
**Homocysteine, µmol/dL**	14 (7)		
**Creatinine, mg/dL**	0.7 (0.2)		
**Estimated GFR, mL/min/1.73 m^2^**	109 (23.5)		
**Proteinuria, mg/day**	345 (90)		
**Uric acid, mg/dL**	4.8 (1.5)		
**Glucose, mg/dL**	77 (7)		
**Lymphocytes, n/µL**	1800 (1000)		
**C-reactive protein, mg/L**	0.33 (0.48)		
**Erythrocyte sedimentation rate, mm/h**	18 (25)		
**Fibrinogen, mg/dL**	282 (85)		

Data are expressed as median (interquartile range), absolute number, or percentages as appropriate. List of abbreviations: SLE, systemic lupus erythematosus; LDL, low-density lipoproteins; HDL, high-density lipoproteins; GFR, glomerular filtration rate; SLEDAI, Systemic Lupus Erythematosus Disease Activity Index; SDI, Systemic Lupus International Collaborating Clinics/American College of Rheumatology Damage Index; ANA, Antinuclear antibody positivity; anti-dsDNA, Anti-double stranded DNA; ENA, Extractable Nuclear Antigens.

**Table 2:** Correlation between markers of arterial stiffness and clinical and laboratory parameters in SLE subjects.

Clinical and Laboratory Parameters	Linear Correlation Coefficient (r)	95% C.I.	*p*
**Univariate Analysis for AoPP**			
**Age**	0.40	0.21 to 0.62	0.02
**Systolic blood pressure**	0.66	0.44 to 0.80	< 0.001
**C-reactive protein**	0.37	0.07 to 0.61	0.03
**Erythrosedimentation rate**	0.34	0.10 to 0.53	0.05
**Daily dose of prednisone**	0.33	0.15 to 0.53	0.05
**SDI score**	0.30	0.12 to 0.44	0.05
**Univariate Analysis for AIx@75**			
**Age**	0.57	0.19 to 0.82	< 0.001
**Lymphocytes**	−0.44	−0.50 to −0.12	< 0.001
**Univariate Analysis for AIx**			
**Age**	0.59	0.21 to 0.85	< 0.001
**Lymphocytes**	−0.34	−0.55 to −0.15	0.05
**Creatinine**	−0.37	−0.62 to −0.12	0.04

List of abbreviations: SLE, Systemic Lupus Erythtematosus; C.I., confidence interval; AoPP, aortic pulse pressure; AIx, augmentation index; AIx@75, normalized to a 75 bpm heart rate AIx; SDI, Systemic Lupus International Collaborating Clinics/American College of Rheumatology Damage Index.

**Table 3:** Multiregression analysis between markers of arterial stiffness and clinical and laboratory parameters in SLE subjects.

Clinical and Laboratory Parameters	Coefficient of Determination (r^2^)	95 % C.I.	*p*
**Multiregression Analysis for AoPP**			
**Age**	0.53	0.20 to 0.89	0.01
**Systolic blood pressure**	0.29	0.12 to 0.45	0.001
**Erythrosedimentation rate**	0.29	0.09 to -0.49	0.005
**Multiregression Analysis for AIx@75**			
**Age**	0.80	0.33 to 1.28	< 0.001
**Multiregression Analysis for AIx**			
**Age**	0.95	0.48 to 1.43	< 0.001

List of abbreviations: SLE, Systemic Lupus Erythtematosus; C.I., confidence interval; AoPP, aortic pulse pressure; AIx, augmentation index; AIx@75, normalized to a 75 bpm heart rate AIx.
